# Fine tuning of trehalose biosynthesis and hydrolysis as novel tools for the generation of abiotic stress tolerant plants

**DOI:** 10.3389/fpls.2014.00147

**Published:** 2014-04-14

**Authors:** Ines Delorge, Michal Janiak, Sebastien Carpentier, Patrick Van Dijck

**Affiliations:** ^1^Department of Molecular Microbiology, VIBLeuven, Belgium; ^2^Laboratory of Molecular Cell Biology, Institute of Botany and MicrobiologyKU Leuven, Leuven, Belgium; ^3^Division of Crop Biotechnics, Department of BiosystemsKU Leuven, Leuven, Belgium

**Keywords:** abiotic stress, trehalose, trehalose-6-phosphate synthase, trehalose-6-phosphate phosphatase, trehalose-6-phosphate, stomata

## Abstract

The impact of abiotic stress on plant growth and development has been and still is a major research topic. An important pathway that has been linked to abiotic stress tolerance is the trehalose biosynthetic pathway. Recent findings showed that trehalose metabolism is also important for normal plant growth and development. The intermediate compound – trehalose-6-phosphate (T6P) – is now confirmed to act as a sensor for available sucrose, hereby directly influencing the type of response to the changing environmental conditions. This is possible because T6P and/or trehalose or their biosynthetic enzymes are part of complex interaction networks with other crucial hormone and sugar-induced signaling pathways, which may function at different developmental stages. Because of its effect on plant growth and development, modification of trehalose biosynthesis, either at the level of T6P synthesis, T6P hydrolysis, or trehalose hydrolysis, has been utilized to try to improve crop yield and biomass. It was shown that alteration of the amounts of either T6P and/or trehalose did result in increased stress tolerance, but also resulted in many unexpected phenotypic alterations. A main challenge is to characterize the part of the signaling pathway resulting in improved stress tolerance, without affecting the pathways resulting in the unwanted phenotypes. One such specific pathway where modification of trehalose metabolism improved stress tolerance, without any side effects, was recently obtained by overexpression of trehalase, which results in a more sensitive reaction of the stomatal guard cells and closing of the stomata under drought stress conditions. We have used the data that have been obtained from different studies to generate the optimal plant that can be constructed based on modifications of trehalose metabolism.

## ABIOTIC STRESS INVOLVES TREHALOSE METABOLISM

In order to discuss stress tolerance in plants, we first should define what stress exactly is since many definitions exist. For instance, Cassells and Curry (2001) propose stress as “an unusual or usual factor of the biotic or abiotic environment modified in such a way that it has the capability of causing injury, disease, or aberrant physiology.” Avoiding unfavorable conditions by physical evasion is generally not an option for plants and therefore other strategies evolved to cope with stress. These include stress avoidance, stress tolerance, or partial escape. For instance during periods of water shortage, a first reaction consists of inhibiting severe water loss, e.g., by closing the stomata (Medrano et al., 2002). Plants that have to face prolonged unfavorable conditions that may be recurrent, will develop some type of tolerance mechanisms to protect them from severe damage, for instance by synthesizing compatible solutes, such as sucrose (Wingler and Roitsch, 2008). Finally, plants can use an escape strategy using dormant seeds that are stress resistant and that will only germinate under favorable conditions. The model plant *Arabidopsis thaliana* is an example of an escaper because it will induce flowering and seed production under stress conditions (Levitt, 1972; Verslues et al., 2006; Lawlor, 2013).

As the current climate change will increase the frequency of unfavorable conditions, the main aim in agricultural research is to develop optimized plants that use efficiently the limited water and nutrient resources during these aberrant conditions in order to maintain growth and preferably, generate a high biomass yield (Passioura, 2012).

In this review, we focus on tolerance mechanisms and more specifically the role of trehalose and its metabolism as putative compatible solute and/or its role in stress tolerance. *In vitro* studies demonstrate clearly the excellent capacity of trehalose to protect membranes and proteins from degradation, where it outcompetes other mono- or disaccharides (Crowe et al., 1998; Magazù et al., 2012). Under *in vivo* conditions, trehalose has been shown to protect cells and organelles from denaturation, but only when present in high concentrations (Crowe, 2007; Chen et al., 2009; Luyckx and Baudouin, 2011). Indeed, in the so-called anhydrobiotic organisms, such as yeast, tardigrades, and some plants, very high trehalose levels (above 10% of the dry weight) help these organisms to survive complete dehydration (Singer and Lindquist, 1998; Iturriaga et al., 2000). This strong increase in trehalose levels is not limited to dehydration conditions, as trehalose levels also accumulate in response to other types of stress, such as heat or oxidative stress (Parrou et al., 1997; Bonini et al., 2004). Consequently, these organisms can survive for extended periods of time in a quiescent state where they are highly resistant to drought, heat, and frost. Upon rehydration, trehalose is completely or partially hydrolyzed, metabolism resumes, and growth is re-initiated. In most plants, however, trehalose levels are far too low in order to have a function as an osmoprotectant. It seems that this function is taken over by sucrose (Salerno and Curatti, 2003). Indeed, during cold and drought stress, plants accumulate sucrose instead of trehalose (Guy et al., 1992). In seeds and pollen, desiccation tolerance is also correlated with sucrose content (Hoekstra and Van Roekel, 1988; Oliver et al., 2005). Sucrose acts as a carrier of energy and carbon in long distance transport. For this purpose, sucrose is more suited due to its high solubility and its higher free energy upon hydrolysis. These findings initially questioned the role of trehalose in stress protection in higher plants.

In plants, trehalose production seemed to be exclusively reserved for stress resistant plants, living in extreme habitats (Gaff, 1971). Based on its excellent characteristics, several attempts to engineer plants that produce more trehalose with the aim to improve stress tolerance and yield under stress conditions have been undertaken in a variety of plant species. Heterologous expression of bacterial or yeast trehalose biosynthesis genes in tobacco, *Arabidopsis*, rice, and potato showed an increased stress tolerance (Holmström et al., 1996). However, by introducing these microbial genes, these plants also showed several aberrant phenotypes, including in several cases decreased plant biomass and altered leaf morphologies (Romero et al., 1997; Goddijn and van Dun, 1999; Garg et al., 2002; Park et al., 2003; Karim et al., 2007). Heterologous expression of *TPS* (trehalose-6-phosphate synthase) genes in plants resulted in opposite phenotypes to plants that were engineered to overexpress trehalose-6-phosphate phosphatase (*TPP*; Schluepmann et al., 2003). This indicated that the level of the intermediate molecule in trehalose biosynthesis, trehalose-6-phosphate (T6P), could be responsible for the aberrant phenotypes. This hypothesis seemed to be true as combined overexpression of a *TPS* and *TPP* gene (either separately, or as a hybrid gene) did not result in aberrant phenotypes (Garg et al., 2002; Karim et al., 2007). A possible explanation for these phenotypes became clear when it was shown, mainly through the plant genome sequencing projects, that all plants contain large trehalose biosynthesis gene families in their genome and that heterologous expression probably interferes with the endogenous T6P levels.

In plants only one pathway for trehalose biosynthesis exists; a two-step process involving TPS and TPP that synthesize and, subsequently, dephosphorylate the intermediate T6P. In *A. thaliana*, there are 11 TPS or TPS-like genes and 10 TPP genes (Thaller et al., 1998; Leyman et al., 2001; Eastmond and Graham, 2003). Interestingly, there is only one trehalase-encoding gene (TRE), which hydrolyzes trehalose into two glucose molecules.

The TPS genes are divided in two classes with class I genes showing homology to the yeast *TPS1* gene and the class II genes showing homology to the yeast *TPS2* gene, which, in yeast encodes for a TPP. In *A. thaliana*, class I consist of four TPS like enzymes, a unique feature among plants which generally only contain one or two class I proteins. From these four genes (AtTPS1–AtTPS4), only AtTPS1 encodes for as active synthase. It contains a plant-specific N-terminal extension (not present in the microbial enzymes; Avonce et al., 2010; Vandesteene et al., 2010). Removal of this domain abolishes a regulatory brake on the enzymatic activity, which emphasizes the importance of a tight regulation of plant endogenous trehalose biosynthesis enzymes (Van Dijck et al., 2002). The class II proteins (TPS5–TPS11) do not show any catalytic activity upon heterologous expression in yeast (Ramon et al., 2009). The TPS domain of these class II proteins lacks the necessary conserved binding sites for uridine-di-phosphate-glucose (UDP-glucose) and glucose-6-phosphate (Glc6-P), which may explain its failure to synthetize T6P. The C-terminal part, however, does contain conserved phosphatase boxes, typical for active TPP enzymes, but remarkably, these proteins do not act as phosphatases. The lack of measurable enzymatic activity and the tissue-specific and developmentally regulated expression patterns of these class II proteins (Zimmerman et al., 2004; Ramon et al., 2009; Vandesteene et al., 2010) suggest a regulatory function, possibly as a sensor for the level of T6P.

The plant *TPP* genes do not show homology with microbial trehalose biosynthesis genes except for the presence of the TPP catalytic phosphatase box domains. The TPP proteins are all active upon heterologous expression in yeast as well as upon expression and purification in *E. coli* (Vandesteene et al., 2012). The large abundance of many active T6P phosphatases is a peculiar fact but most likely necessary for tight regulation of T6P or trehalose levels at the tissue, cellular or subcellular level. Moreover, a tight regulation of these genes is required as even a single knockout can lead to dramatic phenotypes (Satoh-Nagasawa et al., 2006; Van Houtte et al., 2013a).

In wild type plants, there is a strong correlation between the level of T6P and sucrose in a tightly regulated T6P: sucrose ratio. This ratio appears to be critical for plants to maintain their sugar levels as such in certain cell types and/or during certain developmental stages. It is likely that the introduction of constitutively expressed heterologous TPS or TPP genes interferes with this T6P: sucrose proportion by either shifting it higher or lower, respectively (Yadav et al., 2014).

Despite the fact that heterologous expression of TPS and/or TPP genes may result in unwanted phenotypes, in most cases there is also a clear positive effect on stress tolerance. The relationship of trehalose metabolism and stress tolerance is not surprising as *in silico* analysis shows a clear response of many trehalose biosynthesis genes to drought, salt, and cold stress, both in roots as well as in shoots (Iordachescu and Imai, 2008). Moreover, expression of *TPS1* is drought inducible in cotton leaves and roots, and in rice *OsTPP1* and *OsTPP2* have been found to be transiently upregulated by chilling, drought, and abscisic acid (ABA) in both seedling roots and shoots (Pramanik and Imai, 2005; Kosmas et al., 2006).

In the next sections, we will discuss the main findings related to effects of modifying trehalose metabolism and its effect on stress tolerance. In the first paragraph, we will evaluate whether addition (spraying) of trehalose on plants may result in improved stress tolerance. In the following paragraphs, we describe the role of trehalose metabolism for stress tolerance.

## THE ROLE OF EXOGENOUS TREHALOSE IN ABIOTIC STRESS RESPONSE

Plants may encounter external trehalose in cases where plant pathogens or mycorrhizal fungi come into contact with the plant. To understand how plants may react to this, several studies were conducted where trehalose was added to seedlings or adult plants. Trehalose treatment has been shown to induce both biotic and abiotic stress-related genes (Schluepmann et al., 2004). Interestingly, using lower concentrations of trehalose (30 mM instead of 100 mM) together with 1% sucrose showed actually more down-regulated abiotic stress associated genes [e.g., peroxidase 2 (PRXR2)] than upregulated ones (Bae et al., 2005). Apart from the different trehalose concentrations used, these results may also be explained by the use of different DNA microarray providers.

The toxicity of trehalose feeding to plants in high concentrations has been linked to an over-accumulation of T6P, through the regulation of starch metabolism (Schluepmann et al., 2004; Kolbe et al., 2005). These findings have now been linked to a transcription factor bZIP11 (basic region /leucine zipper motif) as bZIP11 overexpression plants show insensitivity toward supplied trehalose (Delatte et al., 2011). Since SnRK1 (sucrose non-fermenting-related kinase-1, a kinase acting as energy sensor) overexpression similarly circumvent growth arrest on trehalose and SnRK1 is postulated to be inhibited by T6P, it might be tempting to speculate a connection between T6P, SnRK1, and bZIP11 to explain the resulting toxicity of trehalose (Zhang et al., 2009; Delatte et al., 2011). Furthermore, feeding trehalose was initially linked to starch metabolism via redox activation of AGPase (adenine-di-phosphate glucose pyrophosphorylase; Kolbe et al., 2005) but more evidence points toward a deteriorated starch breakdown that affects starch levels (Ramon et al., 2007). Indeed, an ethanol-induced overexpression of TPS (OtsA) failed to connect directly elevated T6P with a change in the redox status of AGPase (Martins et al., 2013). Therefore, the change in redox status of AGPase might be an indirect or even independent consequence, possibly in response to sucrose. In fact, studies have shown the connection between T6P and sucrose in *A. thaliana* seedlings (Lunn et al., 2006; Nunes et al., 2013). A recent review by Lunn et al. (2014) specifically deals with the discussion on the connections between T6P, SnRK1, sucrose, and starch.

Furthermore, a disturbed sink/source relationship in seedlings, resulting from the exogenously applied trehalose, leads to abnormal starch accumulation in source organs, which leaves sinks organs, such as roots and developing leaves, out from carbon flow (Wingler et al., 2000). This effect is at least partly ABA-dependent. The uneven balance between starch accumulation, a hampered local starch breakdown and a defect in hexose uptake in roots might explain the toxicity of trehalose (Ramon et al., 2007).

External addition of trehalose can also clearly be beneficial, especially when plants are facing salt stress. Exogenous addition of low levels of trehalose (1–10 mM) to rice plants preserves their root integrity and protects root cells from severe salt stress induced aberrant cell division (Garcia et al., 1997). The protective effect of trehalose could be explained by the preservation of ion pumps, which selectively keep out excess amounts of sodium from the chloroplasts. However, trehalose did not prevent accumulation of salt in the plants cells. Actually, the low accumulation of endogenous trehalose upon salt stress (7 μg/100 mg fresh weight on day 3 of a 1 M NaCl stress) questions its role as osmoprotectant and it is not known whether the sugar remains stable for longer periods of time since no data are presented after three days of stress exposure (Garcia et al., 1997). The protective characteristic of trehalose as elicitor during salt stress is considered as the excellent candidate to preserve lipid bilayer integrity and enzyme functioning during stress conditions (Garcia et al., 1997).

Another protective effect of trehalose is shown in a study where trehalose is linked to the maintenance of seed oil during dehydration. For obvious reasons, the yield and quality of seeds is an important aspect in agriculture for many crops. These characteristics are severely affected by water shortage and can alter seed chemical composition and related qualities such as anti-oxidant activity (Anwar et al., 2006; Ali and Ashraf, 2011). For instance, maize seed oil is valued as one of the best oils worldwide due to its high levels of unsaturated fatty acids, such as oleic and linolenic acid (Ali and Ashraf, 2011). In addition, it also harbors many antioxidants, including flavonoids and phenolics. However, during drought, the level of seed oil is reduced and the contents of oleic and linolenic acid are affected. Spraying a trehalose (30 mM)-Tween 20 solution on the leaves resulted in an improved seed composition and increased antioxidant activity, reflected by a higher level of flavonoids (Ali et al., 2012).

One should take into consideration that externally applied trehalose could be, at least partially, degraded by the trehalase enzyme, residing in the apoplast (Frison et al., 2007). Therefore, protective effects of trehalose should be carefully evaluated, as trehalose might not play the lead role but functions more as an elicitor to induce specific signal transduction pathways, possibly the endogenous trehalose biosynthesis pathway.

## TREHALOSE BIOSYNTHESIS PATHWAY AS POSSIBLE TARGET FOR INCREASING STRESS TOLERANCE

Trehalose biosynthesis is divided into three stages, coinciding with three different enzymatic steps: synthesis of T6P, synthesis of trehalose, and degradation of trehalose. These steps provide interesting tools to modify the levels of T6P or trehalose or both and to evaluate the effect on stress tolerance.

### BIOSYNTHESIS OF T6P BY TREHALOSE-6-P SYNTHASE CLASS I ENZYMES

*Arabidopsis thaliana* is somewhat exceptional by owning more than one class I gene as most plants only contain a single *TPS1* gene, which codes for an active TPS enzyme. *TPS1* is rather low but constitutively expressed in different organs, such as the aerial parts of seedlings, seeds, and hypocotyl (Blazquez et al., 1998). The highest expression is found in sink organs, such as young rosette leaves, flower buds, developing seeds, shoot apical meristem, ripening siliques, and maturing embryos (Zimmerman et al., 2004; Vandesteene et al., 2010). The widespread expression pattern of *TPS1* implicates a unique and essential role throughout the plant’s lifecycle. Interestingly, a knockout of *TPS1* causes embryo lethality and, upon recovery with a transient induction of *TPS1*, shows abnormal growth, small leaves, and retarded development (Eastmond et al., 2002; van Dijken et al., 2004). Clearly, TPS1 is required during seed maturation, but also later on for a correct development and transition from the vegetative to the flowering stage. Altered *TPS1* expression unavoidably coincides with altered T6P levels, which might be the cause of modified responses in flowering transition. Alternatively, T6P joins in a regulatory loop with SnRK1, the sucrose non-fermenting-related kinase-1, a protein involved in energy maintenance (O’Hara et al., 2013). In this way a balance is found to either invest in growth when energy levels are favorable, or to restrain metabolism when energy levels are low.

**FIGURE 1:**
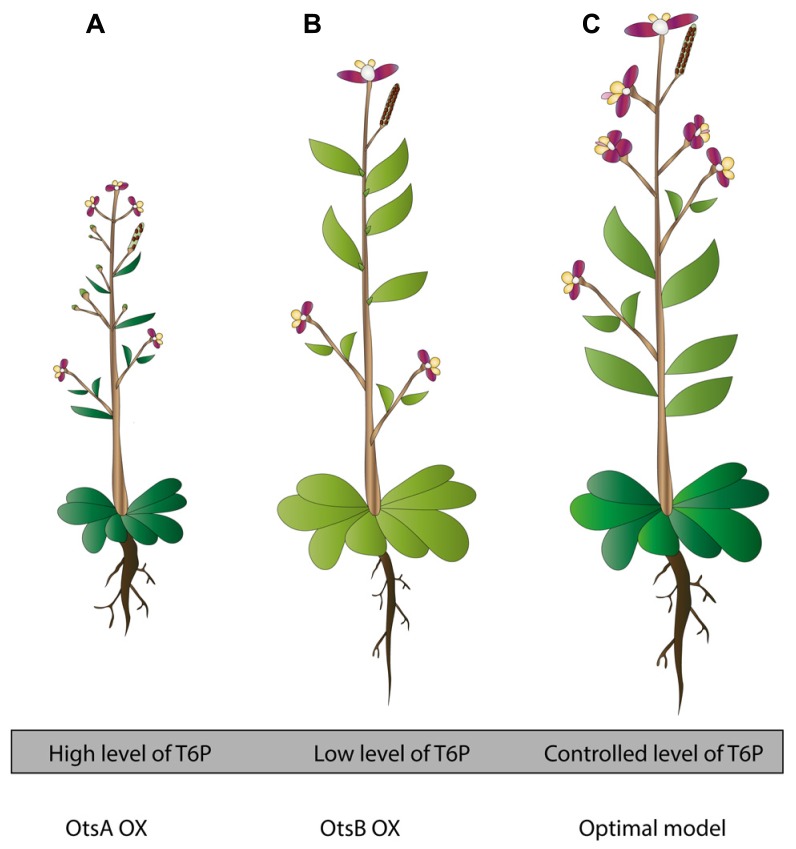
**Transgenic plants in trehalose metabolism: toward an optimized approach for stress tolerant plants. (A)** *OtsA* OX plants contain high levels of T6P. They develop poor seed set, experience reduced apical dominance, flower early and display reduced rosette and darker, lancet shaped leaves. In tobacco *OtsA* OX, root development is disturbed, showing thicker roots. In Chinese cabbage plants *OtsA* OX, thicker roots, well developed lateral roots and extensive root hairs were noticed. In general, these plants are better stress tolerant (based on Goddijn et al., 1997; Park et al., 2003; Schluepmann et al., 2003). **(B)** *OtsB* OX plants contain low levels of T6P. These plants show opposite phenotypes to **(A)**. They develop plenty of seeds, they have distinct apical dominance, flower later, and they have a larger, paler-green rosette (based on Schluepmann et al., 2003). **(C)** Model for optimized transgenic trehalose metabolism plant. This plant has a controlled cellular level of T6P. This optimized model is the result of many factors; use of bifunctional constructs, use of condition specific promoters (for instance stress specific, depending on the destined region where the crop will be grown), use of cell-specific promoters (for instance in stomata, using TRE1 as target gene). In addition, this plant contains optimized constructs, yet unrevealed, connected to abiotic stress tolerance via trehalose metabolism. Ideally, this plant will be tolerant toward a wide range of abiotic stresses, generating high biomass, continuing growth in deteriorated circumstances, generating fertile seeds, and requiring little need for any other kind of maintenance (based on Karim et al., 2007; Van Houtte et al., 2013a).

In an effort to increase trehalose amounts in plants to gain more stress tolerance, research initially turned to TPS enzymes coming from *E. coli* (OtsA) and *Saccharomyces cerevisiae* (Tps1). These attempts were considered fruitful, clearly seen in the enhanced stress tolerance and trehalose biosynthesis but unfortunately led to unexpected phenotypes (**Figure 1**; Holmström et al., 1996). The expression of the *E. coli OtsA* gene in tobacco leads to small, dark-green, lancet-shaped, thick leaves, and a reduced senescence compared with the wild-type control plant (Goddijn and van Dun, 1999; **Figure 1A**). Similar phenotypes were observed when *OtsA* was overexpressed in *A. thaliana* (Schluepmann et al., 2003; **Figure 1A**). Interestingly, overexpressing *OtsA* in *Nicotiana tabacum* connected the increased level of T6P to a higher photosynthetic capacity per unit leaf area and per leaf dry weight, demonstrated by an improved quantum yield of PSII electron transport and CO_2_ assimilation at varying light conditions. However, the resulted higher photosynthetic capacity did not coincide with a higher leaf biomass or growth rate (Pellny et al., 2004).

Altered phenotypes were also apparent in potato and tobacco plants overexpressing *OtsA* or *ScTPS1* which display aberrant root development, but these phenotypes disappeared when plants were grown on soil (**Figure 1**; Goddijn et al., 1997; Yeo et al., 2000). Chinese cabbage plants with *OtsA* overexpressed show thicker taproots, well-developed lateral roots and excessive root hairs (Park et al., 2003). These features are interesting if induced under stress conditions since a high root/shoot ratio is an adaptive characteristic to drought and salinity stress (Roitsch, 1999).

Overexpressing yeast *TPS1* in tomato led to an increase in chlorophyll and starch levels, also in normal conditions, and provided a significant advantage during oxidative, salt, and drought stress (Cortina and Culiáñez-Macià, 2005). Also, when *ScTPS1* was overexpressed in tobacco, an advantage during drought stress conditions was demonstrated despite an abnormal leaf phenotype (Romero et al., 1997). A drought inducible promoter fused to *ScTPS1* in potato caused a slightly improved drought response, due to longer water retention potential and the maintenance of stomatal conductance and net photosynthesis (Stiller et al., 2008). Tight regulation of stomatal movements is very important during drought stress as this regulates optimal water and CO_2_ exchange.

To circumvent aberrant phenotypes, one option explores the overexpressing of endogenous *TPS* genes. Indeed, when overexpressing *AtTPS1* in *A. thaliana*, an enhanced stress tolerance was noticed without any visible morphological abnormalities (Avonce et al., 2004). In addition, transgenic rice, overexpressing its own *TPS1*, improved tolerance toward cold, high salinity, and drought without other significant phenotypic changes. Furthermore, trehalose levels were elevated (up to 40 μg/g fresh weight) and some stress-related genes showed enhanced expression levels, such as ELIP (early light inducible protein) and HSP70 (heat shock protein 70; Li et al., 2011). This indicates that up-regulation of stress-genes are not necessarily linked with aberrant phenotypes, at least not in rice. An alternative approach to circumvent aberrant phenotypes was obtained by expressing the yeast *TPS1* gene in the chloroplast genome instead of the nuclear genome (Lee et al., 2003; Karim et al., 2007).

### TREHALOSE-6-PHOSPHATE SYNTHASE CLASS II GENES

Not much is known about the role of class II proteins during stress. During non-stressed conditions, class II genes are spatially and temporarily differentially expressed (Ramon et al., 2009). Some of these genes have proven their vital role in certain plant processes. TPS6 has been shown to be important for plant architecture, trichome branching, and the shape of epidermal pavement cells (Chary et al., 2008). *TPS5* is expressed in stomata, which might be important during drought stress (Bates et al., 2012). Other class II genes are expressed in leaf primordia, cotyledons, mature root, and root meristematic zone (Birnbaum et al., 2003; Brady et al., 2007). Class II genes do not code for active enzymes and therefore these genes were initially not selected as targets to modify for increased stress resistance. However, this does not exclude a role for class II proteins during stress conditions. For instance, tps5 knockout plants are thermosensitive. During temperature stress TPS5 interacts with MBF1c, a highly conserved transcriptional co-activator (multiprotein bridging factor 1c). MBF1c protein accumulates rapidly during heat stress and functions upstream of other important thermotolerant-related factors such as salicylic acid and ethylene (Suzuki et al., 2008).

In rice, individual overexpression of the class II genes *OsTPS2, OsTPS4, OsTPS5, OsTPS8*, and *OsTPS9* caused a significant tolerance toward cold and salinity stress (Li et al., 2011). Their effect was attributed to the possible complex formation with OsTPS1, which was shown in yeast two-hybrid assays (Zang et al., 2011). Complex formation between class I and class II genes or between class II genes and *TPP* genes may add a possible layer of regulation of the T6P levels similar to the situation in yeast (Bell et al., 1998). In fact, it was previously suggested that such complexes may exist (Geelen et al., 2007). Furthermore, possible post-translational modification of the class II enzymes might occur such as the phosphorylation by SnRK1 and their subsequent binding with 14-3-3 proteins (Harthill et al., 2006).

### BIOSYNTHESIS OF TREHALOSE BY TREHALOSE-6-PHOSPHATE PHOSPHATASE ENZYMES

Whereas *TPS1* seems to be expressed in most plant cells, the dephosphorylation of its product T6P and therefore the production of trehalose seems to be regulated at the cell type level. Indeed, most *TPP* genes of *A. thaliana* are expressed in specific cell types. TPPA can be found in root epidermal cells, pollen, leaves, and anthers whereas *TPPB* and *TPPD* are expressed in roots (Birnbaum et al., 2003; Brady et al., 2007; Vandesteene et al., 2012; Van Houtte et al., 2013a). *TPPG* and *TPPH* show similar expression patterns in root and shoot apical meristem and leaves (TPPG especially in stomata). TPPJ is observed in shoot apical meristem and hydathodes (Vandesteene et al., 2012). The expression of these *TPP* genes is also regulated by environmental conditions, such as light and sugar availability (Van Houtte et al., 2013a). The cell type or tissue-specific expression of the *TPP* genes is also confirmed by the phenotypes observed in single deletion mutants. Deletion of *TPPB* results in larger leaf area as a result of increased cell numbers. TPPA and TPPG may have overlapping functions as only a double knockout of these genes results in a clear hairy root phenotype, which is not observed in the single mutants (Van Houtte et al., 2013a). Also in other species a single *TPP* gene seems to control certain phenotypes. In order to properly control the level of T6P at the cellular level, also the activity of TPS1 should be controlled. This may occur at the level of the N-terminus. One possibility is that TPP enzymes interact with the N-terminal inhibitory domain thereby activating the TPS enzyme.

*RA3*, a maize TPP homolog, is necessary for inflorescence architecture as its knockout causes interesting inflorescence phenotypes (Satoh-Nagasawa et al., 2006).

Plants overexpressing the TPP coming from *E. coli* (*OtsB*) show a slightly enhanced stress tolerance, but display paler, somewhat larger leaves, a characteristic coinciding with a lower rate of net photosynthesis (Pellny et al., 2004; **Figure 1B**). As mentioned above, these are opposite phenotypes to what is observed in plants overexpressing *OtsA*, pointing to the importance of a balanced T6P level in specific cell types for normal growth (**Figure 1**). In order to improve stress tolerance using the *TPP* genes, it will be important to select the appropriate *TPP* gene and express it with a tissue- or cell type-specific promoter or from an abiotic or biotic stress inducible (cell type-specific) promoter.

### BIOSYNTHESIS OF TREHALOSE BY BIFUNCTIONAL CONSTRUCTS

The expression of fusion constructs linking a TPS and TPP domain do not cause aberrant phenotypes, indicating that the T6P that is produced is immediately channeled into trehalose and does not affect cellular metabolism (Goddijn et al., 1997; Garg et al., 2002; Jang et al., 2003; Karim et al., 2007). A bifunctional construct containing *ScTPS1* and *ScTPS2* was introduced in *A. thaliana* under the stress associated rd29A promoter and provided protection against drought, salt, freezing, and heat stress (Miranda et al., 2007). No morphological or growth alterations were observed. Similar results were obtained by introducing the fusion construct of yeast genes in tobacco (Karim et al., 2007). In addition, rice overexpressing both OtsA and OtsB maintained shoot to root K^+^ homeostasis both under stressed and control conditions, a characteristic linked to salt tolerance (Garg et al., 2002). The transgenic rice plants also showed increased root biomass, clearly demonstrated in longer and thicker root phenotypes (Garg et al., 2002).

Root biomass was also increased in maize, inoculated with genetically engineered *Azospirillum brasilense* expressing a fusion *ScTPS1–ScTPS2* construct, although trehalose accumulated in the bacteria, not in the plants (Rodríguez-Salazar et al., 2009).

### BREAKDOWN OF TREHALOSE BY TREHALASE

Most plants seem to only express one trehalase enzyme. In *A. thaliana*, the catalytic domain of this enzyme seems to be localized toward the apoplast which questions its access to the cytoplasmically located substrate. A sensing function to detect external trehalose (coming from pathogenic or beneficiary microorganisms) has been proposed (Müller et al., 2001). In *A. thaliana* trehalase is mainly expressed in floral organs and in maturing siliques, whereas its expression was less observed in stem and roots (Müller et al., 2001; van Dijken et al., 2004; Lopez et al., 2008). Interestingly, trehalase is highly expressed in stomatal guard cells (Van Houtte et al., 2013b). A major surprise was the observation that plants overexpressing trehalase were more drought stress tolerant and knockout of trehalase (Attre1-2 line) resulted in drought stress sensitivity compared to the wild type (Van Houtte et al., 2013b). Interestingly, the higher trehalose levels observed in the KO line counter-intuitively led to the opposite effect since lower trehalose levels in the TRE OX line resulted in better drought stress tolerance. This clearly uncoupled the level of trehalose and drought stress tolerance.

The high expression of trehalase in the stomata connects trehalose metabolism with stomatal regulation (Van Houtte et al., 2013b). The responsible mechanism is not yet known, but seems to involve ABA, as ABA-induced closing of stomata depends on the expression of *AtTRE1* (Van Houtte et al., 2013b). Other studies have also correlated trehalose metabolism to stomata, as ABA induces *TPS1* expression in stomata and *tppg* mutants are insensitive to ABA-induced stomatal closure (Gómez et al., 2010; Vandesteene et al., 2010). In addition, the promoter of *AtTRE1* is predicted to contain a putative binding site for regulation by ABA (Van Houtte et al., 2013a) which is supported by the need for AtTRE1 in ABA-induced closing of the stomata. Stomatal opening and closing is a complex process, which is mediated by many factors, including ABA. Moreover, timing (night/day) and environmental factors (stress/non-stress) also influence stomatal movements. Differential water potential causing opening and closing of stomata is achieved by the exit of different ions, mainly potassium and nitric oxide and malate (MacRobbie, 1983; Desikan et al., 2002; Raschke, 2003). Furthermore, a role for sugar sensing in stomatal movements is very likely as elevated expression levels of hexokinase (HXK) in guard cells causes accelerated stomatal closure and this closure is induced by sugar and ABA, indicating a sucrose-regulated feedback inhibition mechanism (Kelly et al., 2013). Kelly et al. (2013) have demonstrated that during the day sucrose stimulates stomatal closure via HXK and ABA. They hypothesize that the sucrose exported from source cells enters the apoplastic space before it is loaded into the phloem. This apoplastic raise in sucrose reaches the guard cells via the transpiration stream where sucrose is channeled via transporters located in the plasma membrane into the cytosol. Here it is degraded into its hexose components that are recognized by HXK. This recognition by HXK would trigger in association with ABA a reduction in stomatal aperture. We have found that TRE is required for ABA induced stomatal closure (Van Houtte et al., 2013b). We hypothesize that the knockout of TRE1 increases the apoplastic concentration of trehalose and that trehalose might have a higher affinity for the sucrose transporters. By this, trehalose would block the plasma membrane transporters for sucrose transport and hence preventing/lowering the HXK signal and the ABA-mediated stomatal closure. OX of TRE would reduce the apoplastic trehalose content and by this enhance the sucrose transport and stimulate stomatal closure via HXK and ABA. Kelly et al. hypothesized that overexpression of TRE1 leads to an increased glucose monomer concentration that should stimulate the closure response of stomata to ABA (Kelly et al., 2013).

### OPTIMIZING TREHALOSE METABOLISM FOR TRANSGENIC PLANTS

Future research on abiotic stress response should acknowledge the growing impact of a constantly changing environment that causes a huge biomass yield loss even for the most adapted crops (Boyer, 1982; Wang et al., 2003). Those environmental conditions challenge more than ever agricultural industry due to low producing soils and difficult growing conditions. Therefore the optimization of transgenic plants is now considered as one effective strategy to combat expected food shortage. For this reason, we need crops with genetic and epigenetic prosperities already adapted to local environmental conditions. These varieties should have specific characteristics including high leaf area index, little need for fertilization and a broad resistance pattern.

Classical breeding is one way to achieve new cultivars. This method is known for centuries and is widely accepted. However, this approach is time consuming and accelerating demands worldwide for optimized crops question breeding as the optimal way to go.

Genetic modification seems a promising approach but encounters some factors to consider. Optimized tolerant plants have to be well adapted to specific conditions, because these conditions differ even within one country. Another concern involves the severity of stress. Most laboratory approaches apply quite severe stress, which is not always relevant in the field. Moreover, applying stress is not straightforward because stress is a process that most of the time evolves gradually. In many cases even, mild stress is predominantly present before long periods of drought or flooding arrive and plants are already prepared for future damage. In other cases, severe stress does not even occur in long periods, or not even at all. Therefore, although a mild stress approach is more difficult to analyze, it seems the more promising way to go in agricultural research. Furthermore, in nature, different kinds of stresses occur simultaneously and can lead to identical or similar reactions.

This is important, as the mechanisms that stand behind coping with stress (avoidance, tolerance or partial escape) are ultimately not identical. The increased tolerance of certain genetically modified plants under severe conditions do not always show the same phenotype under mild environmental stress circumstances (Skirycz et al., 2011). Moreover, extrapolating results obtained in laboratory conditions to the field might be complex.

Clearly, one should acknowledge multiple factors in the creation of stress tolerant plants but trehalose metabolism could already provide a useful tool to obtain promising results (**Figure 1**). Modification of trehalose metabolism to a plant’s advantage during stress shows that manipulating the endogenous pathway is more reliable than introducing foreign genes that cause aberrant phenotypes (**Figure 1**). Although the combined introduction of both synthase and phosphatase enzymes circumvents these phenotypes, this approach can be quite cumbersome. Traditional methods such as over-expressing or knockout of genes are useful tools to modify endogenous genes and study fundamental principles to reveal interacting factors that come about during stress. However, continuously over-expressing genes could hamper downstream pathways so there is a need for specific promoters that can be switched on and off when desired. The constructed transgenic plants are a good starting point to get fundamental knowledge and examine other unknown influenced pathways that are possible targets for plant engineering in the future (**Figure 1**). Another way to use the trehalose pathway for stress tolerance modification is to investigate in more detail some already well-characterized pathways, such as the trehalase crosstalk with stomatal movements. In respect to drought stress, elucidation of this connection might be a very useful tool.

Finally, the focus must shift toward the elucidation of multiple tolerance mechanisms for more than one abiotic stress that could be engineered in a stepwise process (Debnath et al., 2011). Molecular understanding of the stress perception, signal transduction, and transcriptional regulation of abiotic stress responsive genes may help to engineer tolerance toward multiple stresses. Understanding the molecular mechanism for providing protection against biotic and abiotic stresses may lead to a general master mechanism for stress tolerance.

## CONCLUDING REMARKS

Over the years, trehalose metabolism has widened its impact on plant research clearly seen by many published articles from the last 20 years. Although the research has shifted toward the fundamental aspects of trehalose metabolism and its role during development and growth, there is still some room for modifications toward stress tolerance and resistance in the field. Furthermore it seems that one aspect of research can benefit from the other aspect and vice versa. Therefore, new tools will and can arise that help us to better understand the complex network of trehalose metabolism and pathways during normal and stress conditions.

## AUTHOR CONTRIBUTIONS

All authors contributed to the conception and design of the review. They all contributed to the writing of different parts of the review, and they all finally approved the full text.

## Conflict of Interest Statement

The authors declare that the research was conducted in the absence of any commercial or financial relationships that could be construed as a potential conflict of interest.
